# Supplementary material for: Epigenetic modifier balances Mapk and Wnt signalling in differentiation of goblet and Paneth cells
Source: Life Sci Alliance. 2022 Jan 21;5(4):e202101187. doi: 10.26508/lsa.202101187 (PMC8807877; doi:10.26508/lsa.202101187)
Supplement: Supplementary file 2 [file LSA-2021-01187_TableS1.docx]

### Supplementary Table 1: Primer sequences for ChIP-qPCR.

| ChIP-qPCR primer | sequence (5’-3’) |
| --- | --- |
| GATA6 TSS fwd | GGGTCGCTAGCCAGGTCA |
| GATA6 TSS rev | AGGCAGACAATGAGAGCCGC |
| GATA6_Mll1 fwd | CACAAGCTCTCCGCATTGCC |
| GATA6_Mll1 rev | GCCCCAGAGAGAAATGCAACT |
| TAL1 +70 fwd (Salz *et al*, 2014) | GTGGCCACAAAGCAAGGAAT |
| TAL1 +70 rev (Salz *et al*, 2014) | TCTCTGGAATCTCCAAGGCAA |
