# Supplementary material for: Epigenetic modifier balances Mapk and Wnt signalling in differentiation of goblet and Paneth cells
Source: Life Sci Alliance. 2022 Jan 21;5(4):e202101187. doi: 10.26508/lsa.202101187 (PMC8807877; doi:10.26508/lsa.202101187)
Supplement: Supplementary file 3 [file LSA-2021-01187_TableS2.docx]

### Supplementary Table 2: Primer sequences for qRT-PCR.

| RT-PCR primer | species | sequence (5’-3’) |  |
| --- | --- | --- | --- |
| Axin2_fwd | mouse | AGTCAGCAGAGGGACAGGAA |  |
| Axin2_rev | mouse | CTTCGTACATGGGGAGCACT |  |
| ChgA_fwd | mouse | AGAAGTGTTTGAGAACCAGAGCCC |  |
| ChgA_rev | mouse | TTGGTGATTGGGTATTGGTGGCTG |  |
| EphB2_fwd | mouse | ACTATGAGAAGCAGGAGCTCAGT |  |
| EphB2_rev | mouse | GCTCAAACCCCCGTCTGTT |  |
| EphB3_fwd | mouse | TTCACGACAGTGGGTGACTG |  |
| EphB3_rev | mouse | CGTTGGAGCTGAGTGTCAGA |  |
| Gapdh_fwd (Heuberger *et al*, 2014) | mouse | AAATGGTGAAGGTCGGTGTGAACG |  |
| Gapdh_rev (Heuberger *et al*, 2014) | mouse | TGATGACAAGCTTCCCATTCTCGG |  |
| Gata4_fwd | mouse | AAACGGAAGCCCAAGAACCT |  |
| Gata4_rev | mouse | ACACAGTACTGAATGTCTGGGA |  |
| Gob5_fwd (Heuberger *et al*, 2014) | mouse | TGAAATTGTGCTGCTGACCGATGG |  |
| Gob5_rev (Heuberger *et al*, 2014) | mouse | TGCTGCGAAAGCATCAACAAGACC |  |
| Hes1_fwd | mouse | TGGAAATGACTGTGAAGCACCTCC |  |
| Hes1_rev | mouse | TTGATCTGGGTCATGCAGTTGGC |  |
| Itf_fwd | mouse | TGGGATAGCTGCAGATTACGTTGG |  |
| Itf_rev | mouse | TTTGAAGCACCAGGGCACATTTGG |  |
| Klf4_fwd | mouse | TTAGGCTGTTCTTTTCCGGGGCCACGA |  |
| Klf4_rev | mouse | TTAGGCTGTTCTTTTCCGGGGCCACGA |  |
| Krt20_fwd | mouse | AACTGGCAATGCAGAACCTGAACG |  |
| Krt20_rev | mouse | TAGCATTGTCAATTCGCAGGACGC |  |
| Lyz_fwd (Heuberger *et al*, 2014) | mouse | GCAGCCATACAATGTGCAAAGAGG |  |
| Lyz_rev (Heuberger *et al*, 2014) | mouse | TTTGCCCTGTTTCTGCTGAAGTCC |  |
| Math1_fwd (Shroyer *et al*, 2005) | mouse | ATGCACGGGCTGAACCA |  |
| Math1_rev (Shroyer *et al*, 2005) | mouse | TCGTTGTTGAAGGACGGGATA |  |
| Mll1_fwd | mouse | TGAGCTGCAGATGACTGGTTA |  |
| Mll1_rev | mouse | CCAGAGCATCAGAGGAGAGC |  |
| Mmp7_fwd | mouse | AGGAGTGAACTTCCTGTTTGCTGC |  |
| Mmp7_rev | mouse | TTCTGAATGCCTGCAATGTCGTCC |  |
| Muc2_fwd (Heuberger *et al*, 2014) | mouse | TGTGATGCCAATGACAAGGTGTCC |  |
| Muc2_rev (Heuberger *et al*, 2014) | mouse | ACCACAATGTTGATGCCAGACTCG |  |
| Neurog3_fwd | mouse | ACGCAATTTACTCCAGGCGA |  |
| Neurog3_rev | mouse | GAGGCGCCATCCTAGTTCTC |  |
| Spdef_fwd (Heuberger *et al*, 2014) | mouse | AACATGTATCCCGACGATAGCAGC |  |
| Spdef_rev (Heuberger *et al*, 2014) | mouse | TCAATATCTTTCAGGACCTCGCCC |  |
| Syp_fwd | mouse | TTGGCTTCGTGAAGGTGC |  |
| Syp_rev | mouse | CTGCCGCACGTAGCAAAG |  |
| MATH1_fwd | human | CAGCTGCGCAATGTTATCCC |  |
| MATH1_rev | human | TTGTAGCAGCTCGGACAAGG |  |
| GAPDH_fwd | human | AAGGTGAAGGTCGGAGTCAA |  |
| GAPDH_rev | human | AATGAAGGGGTCATTGATGG |  |
| GATA6_fwd | human | TGCCAACTGTCACACCACAA |  |
| GATA6_rev | human | CATAGCAAGTGGTCTGGGCA |  |
